# Histological characterization of major internal organs in infant common marmosets (*Callithrix jacchus*)

**DOI:** 10.1371/journal.pone.0357273

**Published:** 2026-09-02

**Authors:** Hyeon Ah Kim, Na-Young Lee, Jina Kwak, Hee Jin Choi, Gyeong Min Yoon, Sang Hyeok Seok, Yu Jin Lee, Na Yun Lee, Dae-Yong Kim, Jun Won Park, Byeong-Cheol Kang

**Affiliations:** 1 College of Veterinary Medicine, Department of Laboratory Animal Medicine, Seoul National University, Seoul, Republic of Korea; 2 Department of Experiment Animal Research, Biomedical Research Institute, Seoul National University Hospital, Seoul, Republic of Korea; 3 Seoul National University Hospital Marmoset Model Resource Bank (SNUH-MMRB), Seoul, Republic of Korea; 4 Department of Veterinary Pathology and Research Institute of Veterinary Science, College of Veterinary Medicine, Seoul National University, Seoul, Republic of Korea; 5 Graduate School of Translational Medicine, Seoul National University College of Medicine, Seoul, Republic of Korea; Namik Kemal University: Tekirdag Namik Kemal Universitesi, TÜRKIYE

## Abstract

The common marmoset (*Callithrix jacchus*) is a widely used nonhuman primate model in biomedical research. However, access to infant tissues is limited, and descriptive histological information for early postnatal organ development is needed. We examined a qualitative, descriptive histological overview of five major organs (liver, kidney, spleen, lung and heart) in infant (0–3 weeks) and adult (>12 months) marmosets using hematoxylin and eosin, Ki67 immunohistochemistry, Alcian blue–periodic acid–Schiff, and Sirius red staining. The examined infant livers showed multilayered hepatocyte cords, higher nuclear-to-cytoplasmic ratios, and extramedullary hematopoiesis, and the adult livers exhibited organized hepatic plates and mature stromal structures. The infant kidneys demonstrated active nephrogenesis with immature nephrons, showing progressively more mature glomerular morphology in adults. The spleen displayed small and poorly developed white pulp in infants but fewer, larger, and distinct white pulp structures in adults. The lungs showed thick interalveolarsepta and irregular airspaces during the early postnatal stages, with progressive septal thinning and expanded airspaces in maturity. The infant hearts exhibited loosely packed cardiomyocytes and developing valves, with the adult hearts showing hypertrophied, striated myocardium and compact collagenous valve structures. This study provides descriptive histological information for interpreting developmental features in infant marmosets and supports the use of these data in developmental primate pathology.

## Introduction

Nonhuman primates (NHPs) are widely used in biomedical research where rodent models do not sufficiently reflect primate physiology. Among NHP species, the common marmoset is gaining prominence as a model organism [1–3]. Marmosets offer practical advantages over larger primates, including small body size, high fecundity, rapid maturation, and relatively low maintenance costs [2]. Recent advances in genome editing technologies such as CRISPR/Cas9 have expanded their utility by enabling the generation of transgenic and gene-edited lines for modeling human diseases [4,5]. As a result, common marmosets are used in diverse fields, including neuroscience, regenerative medicine, and aging research [1,6–8]. They share significant anatomical, physiological, and immunological homology with humans [9,10], making them suitable research models.

While comprehensive histological reference data exist for postnatal rodent organ development [11,12], comparable information for NHP infants, including marmosets, remains sparse [13]. Addressing this knowledge gap is important because age-appropriate descriptive histological information is needed to distinguish normal developmental features from pathological alterations in infant primates.

In this study, we systematically examined the histological features of five major organs—the liver, kidney, spleen, lung, and heart—in infant (0–3 weeks) and adult (>12 months) marmosets. Using hematoxylin and eosin (H&E), Ki67, Alcian blue–periodic acid–Schiff (AB–PAS), and Sirius red staining, we examined the histological features of tissue architecture, epithelial and stromal maturation, extracellular matrix organization, and organ-specific developmental patterns in the organs of infant and adult marmosets. This histological characterization provides descriptive information for studies involving postnatal organ development in the common marmoset.

## Materials and methods

### Ethics statement

For this study, six infant marmosets were obtained from breeding colonies maintained at Seoul National University Hospital under an approved protocol (IACUC No. 24-0226). Infant marmoset samples were collected from animals found in a moribund condition following maternal rejection, which commonly occurs in litters of three or more, and were subsequently humanely euthanized under veterinary supervision for animal welfare reasons. In addition, tissues from three adult marmosets were obtained from control animals (saline-treated) used in a separate toxicology study (IACUC No. 20-0170). Cynomolgus monkey tissue samples were obtained from archived tissues collected from a 67-month-old female animal under a separate IACUC-approved study at SNUH (Approval No.: 23-0029-S1A2). These samples were used only as comparative reference tissues to support morphological comparison between primate species in the supplementary figure. This study utilized only previously collected tissues, and no additional animal procedures were conducted.

### Animals

All marmosets used in the experiments were purchased from CLEA Japan, Inc. (Tokyo, Japan). Marmosets were housed at the AAALAC-accredited Marmoset Resource Bank at Seoul National University Hospital (Seoul, Republic of Korea). Animals were kept in stainless steel cages, with bedding changed weekly and cages replaced with clean ones every two weeks. Reverse osmosis (RO) water was provided ad libitum. A commercial diet (Envigo Teklad 8794, Envigo, USA) was offered each morning, supplemented with quail eggs at feeding. The average daily caloric intake per adult animal was approximately 70 kcal. For environmental enrichment, additional food items such as apple, raisins, and bread were provided in the afternoon. The animal room was maintained on a 12-hour light/dark cycle (lights on at 08:00 and off at 20:00), with temperature controlled at 24–28°C and relative humidity maintained at 40–50%. Body weight was recorded weekly, and routine health monitoring—including blood, serum, and fecal microbiological assessments—was conducted annually.

Individuals were categorized into infant and adult developmental stages based on postnatal age. A total of nine marmosets were included in this study, consisting of six infants (PND4-PND22; 4 females, 2 males), and three adults (1–2 years; 3 males).

Mouse tissues were obtained from previously collected normal control animals from an unrelated study conducted under an approved institutional animal care and use protocol (IACUC Approval No. SNU-240217-7). These tissues were used solely for comparative gross anatomical illustration in the Supporting Information and were not included in the histological analyses.

### Anesthesia and Euthanasia

Animals were deeply anesthetized with isoflurane (1–5%) in oxygen (1–4%), as confirmed by the absence of pedal and corneal reflexes and reduced respiratory rate. Animals were then euthanized by exsanguination via the abdominal aorta. All procedures were approved by the Institutional Animal Care and Use Committee of Seoul National University Hospital (SNUH-IACUC; Approval Nos.: No. 24-0226, 20-0170, and 23-0029-S1A2).

### Tissue collection, fixation, and processing

Following euthanasia, necropsy was performed without delay. Major organs, including liver, kidney, spleen, lung, and heart, were rapidly harvested and immersed in 10% neutral buffered formalin (NBF) immediately after collection. Tissues were fixed whole for at least 7 days before trimming, routine processing, and paraffin embedding. Samples from different age groups were processed and stained using matched protocols, including the same fixation and processing protocol.

### Standardized tissue trimming strategy

Gross morphology of marmoset, cynomolgus monkey, and mouse organs was examined to establish a standardized trimming strategy. Based on species-specific anatomy, trimming planes were determined for each organ (S1 Fig). The schematic illustrations shown in S1 Fig were manually created by the authors using ibis Paint X and Microsoft PowerPoint based on original photographs of the specimens obtained during this study. After trimming, tissues were routinely processed and embedded in paraffin (S2 Fig).

Based on species-specific liver anatomy, we adopted a standardized multi-lobe sampling strategy, which is standard practice in pathology studies [14]. Each lobe was dissected individually and sectioned perpendicular to the axis extending from the hilus to maximize histological coverage, resulting in (1) a left lateral lobe slice, (2) a median slice through the gallbladder, (3) a right lateral lobe slice, and (4) a caudate lobe slice (S1A Fig).

A standardized trimming protocol was established for spleen samples (S1B Fig). A single longitudinal section was made along the maximal diameter of each spleen to include both white pulp and red pulp zones, as well as the overlying capsule. This strategy enabled a reliable interspecies comparison of splenic architecture during postnatal development.

Each lung was dissected into individual lobes and prepared for histological sectioning. Lungs were inflated with formalin through intratracheal instillation prior to fixation to preserve alveolar architecture and prevent alveolar collapse [15]. Following inflation and fixation, the lobes were embedded in an anatomically oriented manner and sectioned longitudinally along the bronchial axis (S1C Fig). The selected lobes were trimmed further to ensure adequate visualization of both bronchiolar and alveolar regions within embedding constraints.

A standardized trimming protocol was applied for all kidney samples (S1D Fig). To represent the full corticomedullary gradient, two sectioning planes were defined: (1) a transverse plane at the mid-hilum to capture glomerular zones and medullary rays, and (2) a coronal plane along the longitudinal axis (S1D Fig). The transverse section also visualized the renal pelvis, collecting system, and proximal ureter, enabling a comprehensive evaluation of urinary drainage structures.

A standardized trimming protocol was applied for all heart samples. Hearts were sectioned longitudinally along the long axis to include both atrial and ventricular chambers, the interventricular septum, and cardiac valves within a single section (S1E Fig).

### H&E staining

Tissues from postmortem common marmosets were fixed in 10% NBF immediately after necropsy. Following trimming, the tissues were processed for routine paraffin embedding. Formalin-fixed, paraffin-embedded lung sections (3 μm) were stained with hematoxylin and eosin (H&E). Briefly, sections were deparaffinized in xylene (two changes, 3 min each), rehydrated through graded ethanol (100% ethanol, two changes, 3 min each; 95% ethanol, 3 min; 90% ethanol, 3 min; 70% ethanol, 3 min), and rinsed in distilled water for 5 min. Slides were stained with Harris hematoxylin (Harris Hematoxylin, YD Diagnostics Corp., Yongin, Republic of Korea, S2-5) for 5 min, washed in water for 5 min, differentiated in 0.1% hydrochloric acid solution prepared from 1 N hydrochloric acid (Samchun Pure Chemical Co., Ltd., Pyeongtaek, Republic of Korea, 7647-01-0) for 25 s, and washed again in water for 5 min. Sections were then immersed in 95% ethanol for 1 min and counterstained with eosin-phloxine solution (Labcore Co., Ltd., Seoul, Republic of Korea, 051614−500) for 5 min. After staining, slides were dehydrated in 95% ethanol for 5 sec, followed by 100% ethanol twice for 3 min each, cleared in xylene for 3 min, and kept in xylene until mounting. Histological images were examined using light microscopy.

### Immunohistochemistry

Formalin-fixed, paraffin-embedded tissue sections were deparaffinized in xylene, rehydrated through a graded ethanol series, and subjected to antigen retrieval by heating at 100 °C for 20 minutes in 0.01 mol/L citrate buffer (pH 6.0; C9999, Sigma-Aldrich). Immunohistochemical staining was performed using the ImmPRESS Peroxidase Polymer Detection Kit (Vector Laboratories, Burlingame, CA, USA) following the manufacturer’s instructions. Sections were first blocked with 2.5% horse serum for 60 minutes and then incubated with the primary antibody rabbit anti-Ki67 (Abcam, ab166667; dilution 1:400) for 30 minutes at room temperature. After washing with PBS, the sections were incubated with a polymer-conjugated secondary antibody for 30 minutes, and immunoreactivity was visualized using an ImmPACT DAB peroxidase substrate (SK-4105, Vector Laboratories). Nuclei were counterstained with Gill’s hematoxylin for 45 seconds, followed by rinsing in tap water for 1 minute and differentiation in 0.02% ammonia water for 10 seconds. Final rinsing was performed in distilled water for 1 minute. Negative controls were prepared by omitting the primary antibody. Slides were scanned at 40x magnification using the Pannoramic SCAN digital slide scanner (3D HISTECH, Budapest, Hungary).

### AB–PAS staining

To visualize neutral and acidic mucins, AB-PAS staining was performed on paraffin-embedded tissue sections. Sections were baked at 60 °C for 30 minutes, deparaffinized in xylene, and rehydrated through a graded ethanol series (100%, 95%, 90%, and 70%). Tissues were stained with 1% Alcian blue solution for 15 minutes (prepared in 3% aqueous acetic acid; B8438, Sigma-Aldrich). After rinsing under running tap water and dipping in distilled water, the sections were oxidized in 0.5% aqueous periodic acid for 5 minutes (prepared fresh from P7875, Sigma-Aldrich), followed by rinsing in distilled water. The slides were then incubated in Schiff’s reagent for 10 minutes (PAS solution; Sigma-Aldrich), rinsed three times in distilled water, and counterstained with Gill’s hematoxylin for 45 seconds (HXFHE1LT, Sigma-Aldrich; diluted 1:1 with distilled water). After rinsing in tap water and differentiation in 0.02% ammonia water for 10 seconds, a final rinse in distilled water was performed. Sections were dehydrated through ascending concentrations of ethanol, cleared in xylene, and mounted with a resinous mounting medium.

### Sirius red staining

To visualize collagen fibers in tissue sections, Picro Sirius Red staining was performed using the Picro Sirius Red Stain Kit (Connective Tissue Stain; ab150681, Abcam). Paraffin-embedded sections were first baked at 60 °C for 30 minutes, then deparaffinized with xylene, and rehydrated through a graded ethanol series: 100%, 95%, 90%, and 70% ethanol, followed by distilled water. Slides were incubated with Picro Sirius Red solution at room temperature for 60 minutes to allow thorough staining of the collagen fibers. After incubation, the slides were briefly rinsed twice in 0.5% acetic acid solution to remove excess stain. The sections were then dehydrated in absolute ethanol and cleared in xylene. Finally, the slides were mounted using a synthetic resin-based mounting medium.

### Histological evaluation

Histological evaluation of H&E-stained sections was performed by a board-certified veterinary pathologist (J.W. Park), who was blinded to the age group during slide assessment. All observations were based on qualitative histopathological examination. Organ-specific features were assessed descriptively according to established histological criteria, including tissue architecture, cellular morphology, stromal organization, and developmental characteristics. Representative findings for each organ—including hepatic architecture and extramedullary hematopoiesis in the liver, nephron maturation in the kidney, white pulp development in the spleen, alveolar and bronchial maturation in the lung, and myocardial and valvular maturation in the heart—were evaluated and compared across developmental stages. Histological terminology was standardized according to the Nomina Histologica Veterinaria (NHV, 2017) where applicable.

## Results

### Liver of the infant marmoset

#### Comparative gross morphology.

To account for species-specific anatomical variations in liver structure, we examined the gross morphology of livers from marmosets, cynomolgus monkeys, and mice (S3A Fig). While all three species have a multi-lobed liver architecture with a gallbladder, the organization and number of lobes differ. The common marmoset liver consists of five lobes (left lateral, left central, right central, right lateral, and caudate), with the gallbladder situated in a fossa on the right central lobe [16]. In contrast, the cynomolgus monkey liver comprises four major lobes that can be functionally divided into eight segments, similar to human anatomy [17], while the mouse liver is divided into four lobes with the gallbladder positioned between the median and right lobes. All three species share a conserved caudate lobe and gallbladder structure.

#### Histological characteristics.

**Fig 1:**
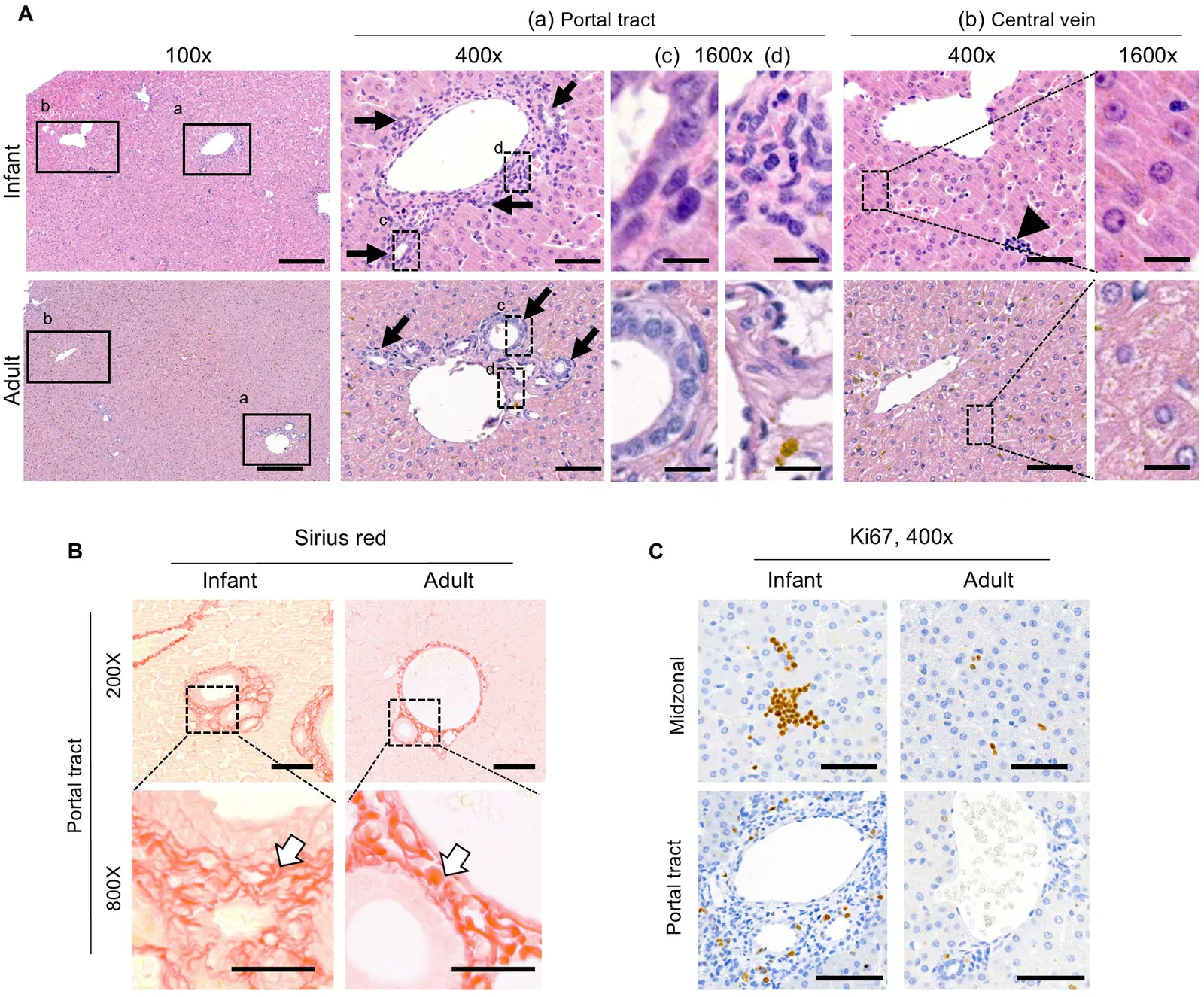
Representative histological and staining-based features of the liver across postnatal development in the marmoset. (A) H&E-stained liver sections at low (100×) and high (400×, 1600×) magnification in infant and adult marmosets. The portal tract (a) and central vein (b) regions are highlighted. Within (a), multiple bile ducts (black arrows) are indicated, one of which is magnified to show its epithelium (c); adjacent basophilic cells exhibiting immature morphology, consistent with primitive stromal or mesenchymal elements (d). The central vein and surrounding parenchyma are shown in (b), with extramedullary hematopoiesis (EMH; arrowheads) and higher magnification revealing hepatocytes. Scale bars: 100×, 200 µm; 400× = 50 µm; 1600× = 12.5 µm. (B) Sirius red staining of liver sections showing perivascular connective tissue (white arrow). Scale bar: 400× = 50 µm; 800× = 25 µm. (C) Ki67 immunostaining of liver sections from infant and adult marmosets. Scale bar: 400× = 50 µm.

As postnatal days progressed, the marmoset livers showed distinct histological changes associated with maturation, reflecting a structural organization toward adult architecture (Fig 1A). In the infant livers, the portal area exhibited histological features of immaturity (Fig 1A), with bile duct profiles lined by a two-cell-thick ductal plate-like epithelium. In the adult livers, the bile ducts were lined by a single layer of cholangiocytes encircling a clear lumen, a morphology consistent with more advanced bile duct organization (Fig 1A). In addition, the hepatocytes in the infant livers were arranged in multilayered cords exceeding two cell layers, contrasting with the single-cell-thick hepatic plates seen in adults (Fig 1A). Although some degree of pleomorphism and nucleus-to-cytoplasmic (N/C) ratio variability is inherent to hepatocytes, the consistently higher N/C ratio and reduced cytoplasmic volume in the infant livers likely reflect postnatal immaturity rather than normal variation (Fig 1A). Extramedullary hematopoiesis (EMH) was observed throughout the hepatic parenchyma within the midzonal and centrilobular regions (Fig 1A). The mesenchymal tissue surrounding the portal vein appeared loose and less fibrous in the infant portal tracts compared to the denser and more organized stroma seen in the adult portal tracts (Fig 1A and 1B). Sirius red staining showed loose and thin collagen fibers around the portal areas in the infant livers, while the adult livers displayed denser and more organized perivascular collagen deposition (Fig 1B).

In the infant livers, multiple Ki-67-positive cells were observed, particularly in regions of EMH identified on H&E sections, suggesting active cellular proliferation within hematopoietic foci (Fig 1C). While the precise lineage of these proliferating cells was not determined, their distribution pattern was consistent with hematopoietic activity (Fig 1C). Additional Ki-67-positive cells were occasionally noted in the portal tracts, which may represent proliferating primitive mesenchymal or stromal cells associated with developing periportal structures (Fig 1C). Only a few scattered Ki-67-positive cells were observed in the adult livers, primarily within stromal rather than parenchymal compartments, indicating markedly reduced proliferative activity (Fig 1C).

Infant marmoset livers exhibited active hematopoietic function and immature structural features, including a high N/C ratio, disorganized hepatocyte cords, and loosely arranged connective tissue. These findings were accompanied by age-associated morphological differences toward adult hepatic architecture, including reduced extramedullary hematopoietic activity, organized hepatic plates, and more developed stromal structures.

### Kidney of the infant marmoset

#### Comparative gross morphology.

To address interspecies anatomical differences in kidney morphology, we examined the gross anatomy of kidneys from marmosets, cynomolgus monkeys, and mice (S4A Fig). Although all three species have a unipapillary kidney structure with conserved corticomedullary architecture, their kidneys show generally similar relative proportions despite minor interspecies variation [18–20].

#### Histological characteristics.

**Fig 2:**
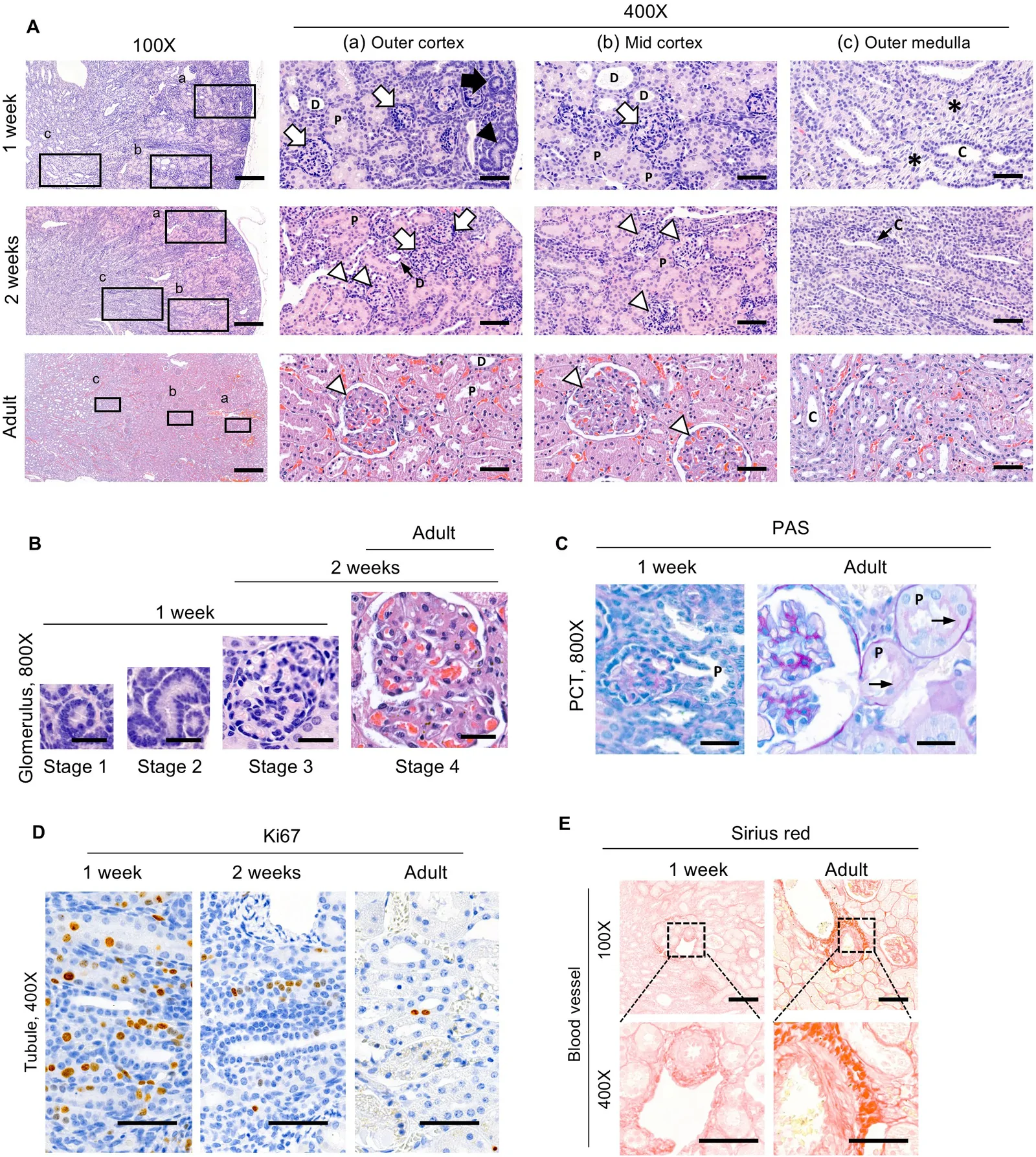
Representative histological and staining-based features of the kidney across postnatal development in the marmoset. (A) H&E-stained kidney sections at low (100×) and high (400×) magnification from three developmental stages (1 week, 2 weeks, and adult). Immature nephron structures in the outer cortex at 1 week are indicated as stage 1 (black arrow), stage 2 (black arrowhead), and stage 3 (white arrow). Stage 4 nephron structures in adults are indicated (white arrowhead). Proximal convoluted tubules (P), distal convoluted tubules (D), collecting ducts (C), and intertubular connective tissue (*) are shown. Scale bars: 100× = 200 µm; 400× = 50 µm. (B) Sequential nephron developmental stages (S1–S4) observed in infant and 2-week kidney sections. Scale bars: 800× = 25 µm. (C) PAS-stained kidney sections showing proximal convoluted tubules from infant and adult marmosets. Scale bars: 800× = 25 µm. (D) Ki67 immunostaining of tubular structures in kidneys from infant and adult marmosets. Scale bars: 400× = 50 µm. (E) Sirius red staining of kidney sections showing perivascular collagen in infant and adult samples. Scale bars: 100× = 200 µm; 400× = 50 µm.

The marmoset kidneys exhibited distinct age-dependent histological changes in nephron maturation and tubular organization. The infant kidneys (one week postnatal, PND6; two weeks postnatal, PND15) featured immature nephrons with undifferentiated glomerular and tubular structures (Fig 2A). The outer cortex showed densely packed immature tubules, confirming active nephrogenesis (Fig 2A). The surrounding mesenchyme was loose with low fibrous organization of the interstitial matrix (Fig 2A).

In the renal cortex, the proximal convoluted tubules (PCTs) in adults possessed a prominent brush border, giving the apical surface a fuzzy or irregular appearance (Fig 2A and S5 Fig). In contrast, in the immature kidneys, the PCT lumen was relatively narrow and not well defined (Fig 2A and S5 Fig). Distal convoluted tubules (DCTs) were lined by more cuboidal epithelial cells, with a defined luminal contour during the early postnatal stages (Fig 2A and S5 Fig).

In the medulla, the collecting ducts were lined with cuboidal epithelial cells. At one week of age, these cells had relatively large basophilic nuclei, and the ducts showed irregular luminal contours within the outer medulla (Fig 2A and S5 Fig). As the kidneys matured, the epithelial lining became more uniform (Fig 2A and S5 Fig). At two weeks of age, the amount of primitive loose connective tissue had decreased compared to one week of age, resulting in reduced spacing between adjacent tubules (Fig 2A and S5 Fig).

Immature nephrons correspond to distinct developmental stages (S1–S4) that can be sequentially observed within the nephrogenic zone adjacent to the renal capsule (Fig 2B). At the earliest S1 stage, nephrons appear as ovoid condensations of metanephric mesenchyme in the superficial cortex (Fig 2B). As development proceeds to the S2 stage, the structures adopt an S-shaped morphology and are lined by undifferentiated columnar epithelial cells surrounding a primitive lumen (Fig 2B). In the S3 stage, cuboidal podocyte precursors and parietal epithelial cells become distinguishable, although the Bowman’s space remains narrow or absent (Fig 2B). In the S4 stage, nephrons display flattened podocytes and parietal cells with an expanded Bowman’s space, indicating progressive glomerular maturation (Fig 2B). At one week of age, most nephrons are in the S1 to S3 stages, and by two weeks, the S1 and S2 stages are rarely observed and the S3 to S4 stages predominate, reflecting the rapid decline in nephrogenic activity and concurrent epithelial differentiation (Fig 2A).

In the PAS-stained sections, the apical surface of PCT epithelial cells in one-week-old infant kidneys showed little to no staining (Fig 2C). In contrast, the adult kidneys exhibited a well-developed brush border, visible as a distinct bright magenta band along the luminal edge of the PCTs (Fig 2C).

Ki-67 immunohistochemistry showed numerous Ki-67 positive tubular epithelial cells, indicating ongoing renal development and high mitotic activity (Fig 2D). Only a few Ki-67 positive cells were observed in the adult kidneys compared with the infant kidneys, consistent with the morphologically mature appearance of the renal tissue (Fig 2D). Sirius red staining showed differences in the maturation of the interstitial matrix, with sparse and loosely arranged collagen fibers in infants progressing to more organized perivascular and interstitial fibrosis in adults, reflecting vascular tissue maturation (Fig 2E).

These findings demonstrate that infant marmoset kidneys exhibit ongoing nephrogenesis and immature tubular and glomerular structures, while adult marmoset kidneys display completed nephron development with well-organized tubules and matured glomeruli, reflecting structural renal maturation.

### Spleen of the infant marmoset

#### Comparative gross morphology.

To address interspecies anatomical differences in spleen morphology, we compared the gross anatomy of spleens from marmosets, cynomolgus monkeys, and mice (S6A Fig). While all three species exhibited a conserved internal organization composed of white pulp, red pulp, and fibrous capsules, differences in relative size and gross appearance were evident. Although the overall splenic architecture was conserved across species, cynomolgus monkeys and mice showed similar relative spleen sizes, whereas marmosets displayed roughly half the proportion [18,20,21]. In cross-sectional views, the marmoset and cynomolgus spleens exhibited relatively sparse and less distinct white pulp nodules compared to that of the mice, whose spleens showed more abundant and prominent white pulp visible even at the gross level (S6B Fig). This reflects species-specific differences in splenic white pulp organization; mice display higher follicular density per unit area of splenic tissue compared to larger primates and humans [22].

#### Histological characteristics.

**Fig 3:**
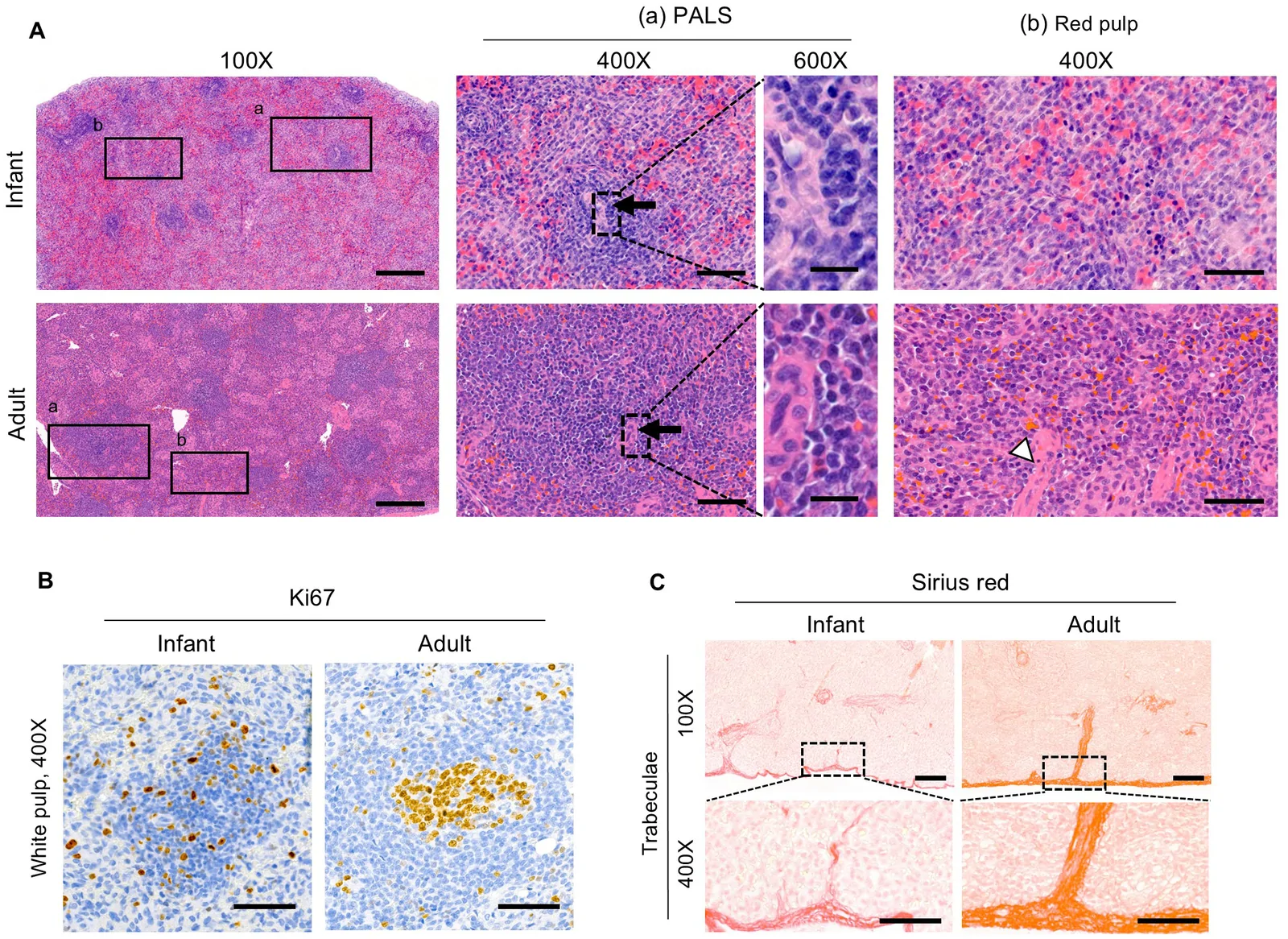
Representative histological and staining-based features of the spleen across postnatal development in the marmoset. (A) H&E-stained spleen sections from infant and adult marmosets at low (100×) and high (400×) magnification. White pulp structures and periarteriolar lymphatic sheaths (PALS) surrounding the central arteriole are indicated (black arrows). Nucleated erythroid precursors in the red pulp are shown. Trabeculae are indicated (white arrowheads). Scale bars: 100× = 200 µm; 400 × , 600× = 50 µm. (B) Ki67 immunostaining of spleen sections from infant and adult marmosets. Scale bars: 400× = 50 µm. (C) Sirius red staining of spleen sections showing collagen distribution within trabeculae and perivascular regions in infant and adult samples. Scale bars: 100× = 200 µm; 400× = 50 µm.

In infant marmoset spleens, EMH was minimal, and periarteriolar lymphoid area, morphologically consistent with developing periarteriolar lymphatic sheaths (PALSs), appeared as small lymphoid aggregates surrounding the central arterioles (Fig. 3A). As the spleen matured, these periarteriolar lymphoid areas appeared more expanded and better defined, suggesting progressive morphological organization of the white pulp (Fig 3A). Consistent with structural maturation, EMH was rarely observed in the adult spleens; splenic arterioles were surrounded by broad areas of lymphocytes, a finding morphologically consistent with well-developed periarteriolar lymphoid tissue (Fig 3A). The red pulp exhibited well-formed trabeculae along with pale eosinophilic stromal tissue (Fig 3A). Relatively few Ki-67 positive cells were observed in the white pulp during infancy, while the adult spleen displayed well-defined germinal centers with high Ki-67 positivity (Fig 3B). Sirius red staining showed sparse collagen deposition around the arteries and trabeculae in the infant spleens, with a more organized and dense fibrous architecture observed in the adult spleens (Fig 3C).

These findings indicate that the marmoset spleen undergoes gradual immunological compartmentalization and stromal remodeling postnatally, with increased white pulp size and number and progressive stromal maturation progressing toward a mature lymphoid structure.

### Lung of the infant marmoset

#### Comparative gross morphology.

To address interspecies anatomical differences in lung morphology, we compared the gross anatomy of lungs from marmosets, cynomolgus monkeys, and mice (S7A Fig). The number and arrangement of lobes varied across species. The marmoset lung comprises left cranial and caudal lobes and right cranial, middle, caudal, and accessory lobes. The cynomolgus monkey lung consists of two lobes on the left (cranial and caudal) plus an accessory lobe, and three lobes on the right (cranial, middle, and caudal), while the mouse lung features a single left lobe and four right lobes (cranial, middle, caudal, and accessory).

#### Histological characteristics.

**Fig 4:**
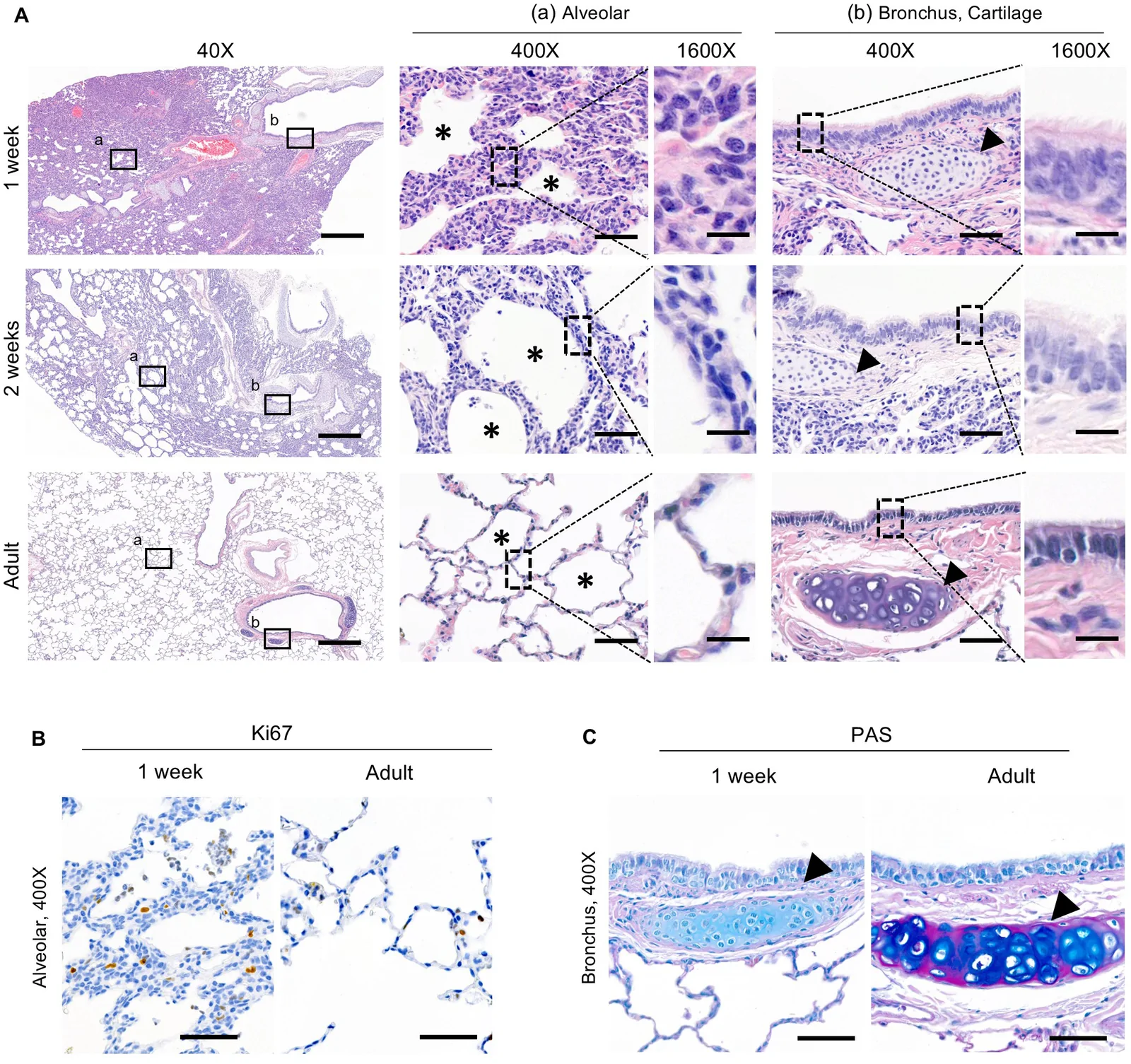
Representative histological and staining-based features of the lung across postnatal development in the marmoset. (A) H&E-stained lung sections at low (40×) and high (400 × , 1600×) magnification from 1-week, 2-week, and adult stages. Alveolar region (a) highlights airspaces (asterisks), with interalveolar septa magnified. Bronchus (b) shows cartilage (arrowheads), with terminal bronchioles at higher magnification. Scale bars: 40× = 200 µm; 400× = 50 µm; 1600× = 12.5 µm. (B) Ki67 immunostaining of lung sections from infant and adult marmosets. Scale bars: 400× = 50 µm. (C) PAS-stained bronchial cartilage from infant and adult marmosets, with extracellular matrix indicated (arrowheads). Scale bars: 400× = 50 µm.

The histological analysis of lung development revealed distinct morphological changes across the marmoset postnatal stages (Fig 4A). Infant lungs (one week postnatal, PND6) showed thick interalveolar septa and irregular alveolar spaces, reflecting a structurally immature alveolar architecture (Fig 4A). The alveolar walls appeared thick and cellular, likely composed of immature epithelial and mesenchymal components.

By two weeks of age (PND15), the interalveolar septa had become thinner and more defined, with increasing airspace expansion and reduced cellularity in the interstitium (Fig 4A). In the adult lungs, the alveolar walls were uniformly thin and consisted of a single layer of squamous epithelial cells supported by a minimal interstitial matrix (Fig 4A). The alveolar spaces (indicated by asterisks in Fig 4A) appeared expanded and well defined compared to those in the infant lungs. This progressive thinning and simplification of the interalveolar septa across postnatal development reflects the process of alveolarization, which is a key morphogenetic event in primate lung maturation (Fig 4A). Through this process, primitive sac-like structures transition into mature alveoli with an expanded surface area and attenuated septal thickness, thereby completing the establishment of adult alveolar architecture (Fig 4A).

The bronchus is initially lined with ciliated, basophilic pseudostratified epithelium, which gradually transforms into a simple cuboidal to columnar epithelium (Fig 4A). In infants, cartilage is composed of loosely organized chondroblasts with large round nuclei and a pale immature matrix lacking distinct lacunae (Fig 4A). As maturation proceeds, the cartilage becomes more structured, with well-defined chondral lacunae containing smaller, differentiated chondrocytes, and a denser, eosinophilic extracellular matrix (Fig 4A).

Numerous Ki-67 positive cells were observed in the alveolar epithelial cells and the bronchus epithelium of infant marmosets, consistent with active postnatal lung remodeling and growth (Fig 4B). The adult lungs showed fewer Ki-67-positive cells, consistent with reduced cellular proliferation in morphologically mature lung tissue (Fig 4B).

PAS staining highlighted distinct changes in cartilage composition and structure (Fig 4C). The adult lungs displayed well-demarcated cartilage areas with more intense PAS positivity, suggesting progressive cartilage differentiation and maturation of the glycoprotein-rich matrix (Fig 4C).

These findings indicate age-associated morphological maturation of the postnatal marmoset lung, characterized by thinner interalveolar septa, expanded alveolar spaces, and progressive organization of cartilaginous airway structures.

### Heart of the infant marmoset

#### Comparative gross morphology.

To enable a reproducible assessment of cardiac structure, we compared the gross morphology of hearts from common marmosets, cynomolgus monkeys, and mice (S8A Fig). All specimens displayed conserved cardiac chambers, including bilateral atria and ventricles. However, significant interspecies differences in relative heart size were observed, with primate hearts comprising a substantially smaller proportion of body weight compared with mice [20,23,24].

#### Histological characteristics.

**Fig 5:**
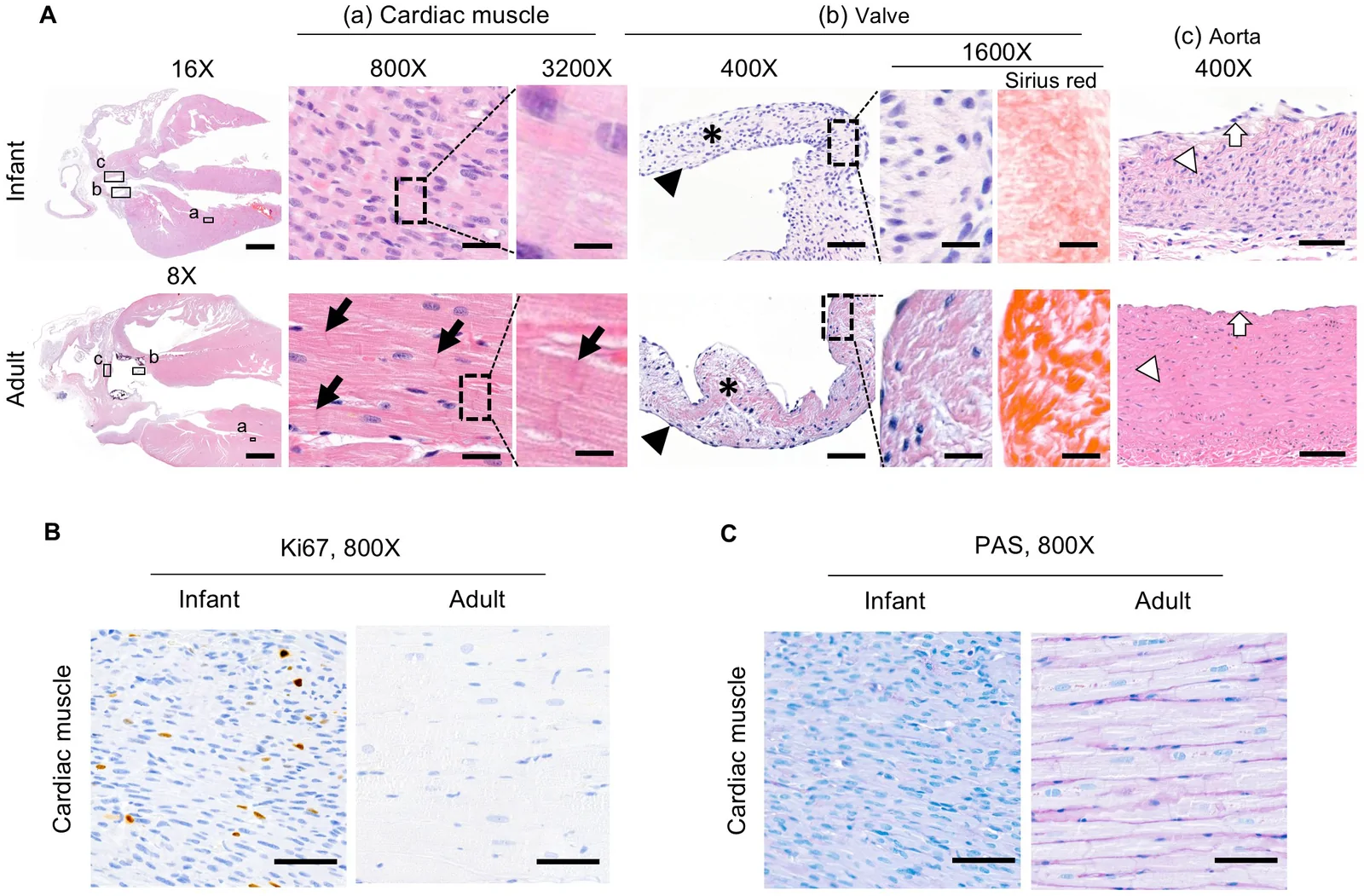
Representative histological and staining-based features of the heart across postnatal development in the marmoset. (A) H&E- and Sirius red–stained heart sections from infant and adult marmosets at low (8x, 16x) and high magnification (400 × , 800 × , 1600 × , 3200×). Cardiac muscle regions (a) show cardiomyocyte striations, intercalated discs (black arrow), nuclei, and fiber orientation. Valve structures (b) display connective tissue components (asterisks) and surface endothelial cells (arrowheads). Sirius red staining highlights collagen distribution within valve leaflets. The aortic wall (c) shows elastic lamellae (white arrows) and tunica intima (white arrowhead). Scale bars: 8x = 2000 µm; 16x = 1000µm; 400× = 50 µm; 800× = 25 µm; 1600× = 12.5 µm; 3200× = 6.25 µm. (B) Ki67 immunostaining of cardiac tissue from infant and adult marmosets. Scale bars: 800× = 25 µm. (C) PAS-stained cardiac muscle from infant and adult marmosets showing perimyocyte extracellular matrix labeling. Scale bars: 800× = 25 µm.

Marmoset postnatal heart development showed distinct structural and cellular maturation through histological and histochemical analyses (Fig 5A). Histologically, the infant hearts featured a high nuclear density with loosely arranged cardiomyocytes and relatively thin myocardial layers (Fig 5A). Cardiomyocyte striation patterns were indistinct with poorly defined intercalated discs, suggesting immature sarcomere organization (Fig 5A). With growth, cardiomyocytes became hypertrophied with increased cytoplasmic volume and distinct striation patterns with clear intercalated discs (black arrow), indicating myofibrillar maturation (Fig 5A).

As the cardiac valves matured, they became progressively less cellular, with a marked reduction in resident cell density (Fig 5A). The extracellular matrix (asterisk), initially composed of loose collagen, transitioned into denser and more intensely eosinophilic collagen bundles in adults (Fig 5A). Additionally, the surface endothelial cells (black arrowhead), which appeared plump in the infant hearts, became progressively flattened with age, reflecting structural maturation (Fig 5A). Sirius red staining showed that the heart valves in infants exhibited faint staining with loosely and sparsely arranged collagen fibers (Fig 5A). In comparison, the adult valves exhibited stronger staining with densely packed and well-organized collagen bundles, reflecting extracellular matrix maturation and structural reinforcement with age (Fig 5A).

In the aorta, the elastic laminae (white arrowhead) appeared thin and undulating in the infant hearts but became progressively thicker with age (Fig 5A). Medial smooth muscle cells exhibited large round nuclei with a high N/C ratio in the early stages, while their nuclei appeared smaller in the adult hearts, reflecting cellular maturation (Fig 5A). The endothelial lining of the tunica intima (white arrow), plump in the early postnatal period, gradually flattened with age, consistent with vascular structural maturation (Fig 5A).

In the cardiac muscle, Ki-67 immunostaining revealed numerous Ki-67 positive cells in the infant hearts, with fewer Ki-67 positive cells observed in the adult hearts, consistent with the postnatal exit of cardiomyocytes from the cell cycle (Fig 5B). PAS staining showed weak reactivity in the perimyocyte connective tissue of the infant hearts, whereas more intense PAS staining was observed around the cardiac muscle fibers (Fig 5C).

These findings reveal that marmoset cardiac development is characterized by a postnatal decrease in myocyte nuclear density, progressive cardiomyocyte hypertrophy, and the maturation of myofibrillar structures and cardiac valves, establishing adult myocardial architecture.

## Discussion

By examining five major organs in infant and adult marmosets, this study provides a descriptive histological overview characterizing the structural differences associated with postnatal development in this species. We identified organ-specific features of postnatal maturation, including prolonged extramedullary hematopoiesis in the liver, alveolar remodeling in the lung, ongoing nephrogenesis in the kidney, progressive immunological compartmentalization in the spleen, and cardiomyocyte hypertrophy with myofibrillar maturation in the heart.

From a developmental pathology perspective, distinguishing normal maturational features from pathological alterations is essential when evaluating tissues from infant animals. During early postnatal development, several histological findings often associated with disease in adults may also occur as part of normal organ maturation. For instance, hepatic extramedullary hematopoiesis and increased Ki67 labeling reflect active hematopoietic and proliferative activity during fetal and early postnatal life, but similar features can also be observed under pathological conditions such as inflammation, regenerative responses, or systemic stress [25]. Likewise, thickened interalveolarsepta in the developing lung and immature glomerular structures in the kidney represent expected developmental stages, but comparable changes may also indicate injury or disease, depending on the context [26]. Recognizing these features as developmental rather than pathological is critical for accurate histopathological interpretation in infant primates, where baseline reference data remain limited; thus, our study provides a comparative framework to support developmental pathology assessments in early life.

To contextualize these findings within the broader framework of primate development, we compared the developmental patterns of the marmoset with those of widely used rodent models. Altricial rodents such as mice are born neurologically and physiologically immature [27], with external features including sealed eyes and ears. Although external development occurs rapidly postnatally, histological examination shows organs characteristic of developmental immaturity, with minimal organ structure differentiation in the early postnatal period [27,28]. In contrast, marmosets, while neurally altricial relative to other primates, are born with relatively advanced external maturity at approximately 8% of adult body weight [29], with eyes open and mobile [30]. However, despite their external precocial appearance, the infant marmosets in this study showed persistent structural immaturity in multiple organ systems during early postnatal life. The mixed immature–mature histologic profiles observed in the infant marmosets, as opposed to mouse models, are broadly consistent with previous descriptions of human neonatal tissues, including sustained hepatic hematopoiesis and partially developed biliary structures [31]. This developmental pattern supports the potential utility of the marmoset as a model for studies of postnatal organ development.

This study has several limitations. First, the sample size was modest, and the distribution of sex differed between the infant and adult groups, which may limit the generalizability of the findings. Second, the study was based on a cross-sectional evaluation of archived tissue specimens, which precluded longitudinal assessment of postnatal organ development within the same individuals. Third, the histological, immunohistochemical, and histochemical evaluations were qualitative and were not supplemented by quantitative image analysis or morphometric measurements. Finally, lineage-specific markers were not included to further characterize proliferating or immature cell populations. Nevertheless, the consistent methodology applied across all specimens allowed a systematic qualitative comparison of postnatal histological features, providing a useful descriptive overview of organ maturation in the common marmoset.

In conclusion, this study provides an overview of the early postnatal histological features of five major organs of the common marmoset, offering reference data that reflect normal tissue characteristics during the infant period. By characterizing developmental features across postnatal stages, this work supports more accurate interpretation of findings of studies using young marmosets, enhances the understanding of primate organ maturation, and contributes to the broader utility of the marmoset as a physiologically relevant model in biomedical research.

## Supporting information

S1 Fig Standardized tissue trimming protocols for major internal organs across species. Left: marmoset, middle: cynomolgus monkey, right: mouse. (A) Schematic liver diagrams of the species-specific liver trimming strategy. White dashed lines indicate these planes, which were adapted to each species’ lobe proportions. (B) Schematic spleen diagrams illustrating the trimming strategy. Each spleen was sectioned along its longitudinal section to include representative areas of the capsule, red pulp, and white pulp. (C) Schematic lung diagrams illustrating species-specific trimming strategies. White dashed lines indicate these planes, which were adapted to each species’ lobe proportions. (D) Schematic kidney diagrams illustrating the trimming strategy, which was applied uniformly across all three species. Kidneys were sectioned along the coronal plane to expose both the cortex and the medulla, with standard sampling at two representative levels: transverse and coronal. (E) Schematic heart illustration of the trimming strategy which was applied uniformly across all three species. Hearts were sectioned longitudinally along the long axis to include the ventricular chambers, interventricular septum, and portions of the atria.
(JPG)

S2 Fig Representative paraffin embedding of trimmed tissues following standardized sectioning. Left: marmoset, middle: cynomolgus monkey, right: mouse. (A) Liver tissues embedded in paraffin blocks following trimming and tissue processing. White arrowhead = gallbladder; dashed box = mouse liver. (B) Paraffin embedding of trimmed spleen sections. Left dashed box = marmoset spleen; middle dashed box = cynomolgus monkey spleen; right dashed box = mouse spleen. (C) Paraffin-embedded lung lobes from each species. Dashed box = mouse lung. (D) Paraffin embedding of trimmed kidney sections. Dashed box = mouse kidney. (E) Paraffin embedding of trimmed heart tissues.
(JPG)

S3 Fig Representative liver gross morphology. Left: marmoset, middle: cynomolgus monkey, right: mouse. (A) Dorsal and ventral views of the liver in adult marmoset, cynomolgus monkey, and mice. Scale bars: 2 cm.
(JPG)

S4 Fig Representative kidney gross morphology. Left: marmoset, middle: cynomolgus monkey, right: mouse. (A) Gross appearance of the kidneys from adult marmosets, cynomolgus monkeys, and mice. Scale bars: 1 cm.
(JPG)

S5 Fig Representative histological features of renal tubules across postnatal development in the marmoset. (A) Proximal convoluted tubule (P), (B) distal convoluted tubule (D), and (C) collecting duct (C) are shown at 1600× magnification in kidneys from 1-week-old, 2-week-old, and adult marmosets. Scale bars = 20 µm.
(JPG)

S6 Fig Representative spleen gross morphology. Left: marmoset, middle: cynomolgus monkey, right: mouse. (A) Gross morphology of spleens from adult marmosets, cynomolgus monkeys, and mice, showing species-specific differences in size, shape, and surface contour. Scale bars: 1 cm.
(JPG)

S7 Fig Representative lung gross morphology. Left: marmoset, middle: cynomolgus monkey, right: mouse. (A) Dorsal and ventral gross views of the lungs in adult marmosets, cynomolgus monkeys, and mice. Scale bars: 1 cm.
(JPG)

S8 Fig Representative heart gross morphology. Left: marmoset, middle: cynomolgus monkey, right: mouse. (A) Gross images of the heart from adult marmosets, cynomolgus monkeys, and mice, demonstrating interspecies variation in organ size and shape. Scale bars: 1 cm.
(JPG)
